# Associations of Depression, Anxiety, Worry, Perceived Stress, and Loneliness Prior to Infection With Risk of Post–COVID-19 Conditions

**DOI:** 10.1001/jamapsychiatry.2022.2640

**Published:** 2022-09-07

**Authors:** Siwen Wang, Luwei Quan, Jorge E. Chavarro, Natalie Slopen, Laura D. Kubzansky, Karestan C. Koenen, Jae Hee Kang, Marc G. Weisskopf, Westyn Branch-Elliman, Andrea L. Roberts

**Affiliations:** 1 Department of Nutrition, Harvard T.H. Chan School of Public Health, Boston, Massachusetts; 2 Department of Environmental Health, Harvard T.H. Chan School of Public Health, Boston, Massachusetts; 3 Department of Epidemiology, Harvard T.H. Chan School of Public Health, Boston, Massachusetts; 4 Channing Division of Network Medicine, Department of Medicine, Brigham and Women’s Hospital, Harvard Medical School, Boston, Massachusetts; 5 Department of Social and Behavioral Sciences, Harvard T.H. Chan School of Public Health, Boston, Massachusetts; 6 Psychiatric Neurodevelopmental Genetics Unit, Department of Psychiatry, Massachusetts General Hospital, Boston, Massachusetts; 7 VA Boston Healthcare System, Department of Medicine, Section of Infectious Diseases, Boston, Massachusetts; 8 Harvard Medical School, Boston, Massachusetts

## Abstract

**Importance:**

Few risk factors for long-lasting (≥4 weeks) COVID-19 symptoms have been identified.

**Objective:**

To determine whether high levels of psychological distress before SARS-CoV-2 infection, characterized by depression, anxiety, worry, perceived stress, and loneliness, are prospectively associated with increased risk of developing post–COVID-19 conditions (sometimes called long COVID).

**Design, Setting, and Participants:**

This prospective cohort study used data from 3 large ongoing, predominantly female cohorts: Nurses’ Health Study II, Nurses’ Health Study 3, and the Growing Up Today Study. Between April 2020 and November 2021, participants were followed up with periodic surveys. Participants were included if they reported no current or prior SARS-CoV-2 infection at the April 2020 baseline survey when distress was assessed and returned 1 or more follow-up questionnaires.

**Exposures:**

Depression, anxiety, worry about COVID-19, perceived stress, and loneliness were measured at study baseline early in the pandemic, before SARS-CoV-2 infection, using validated questionnaires.

**Main Outcomes and Measures:**

SARS-CoV-2 infection was self-reported during each of 6 monthly and then quarterly follow-up questionnaires. COVID-19–related symptoms lasting 4 weeks or longer and daily life impairment due to these symptoms were self-reported on the final questionnaire, 1 year after baseline.

**Results:**

Of 54 960 participants, 38.0% (n = 20 902) were active health care workers, and 96.6% (n = 53 107) were female; the mean (SD) age was 57.5 (13.8) years. Six percent (3193 participants) reported a positive SARS-CoV-2 test result during follow-up (1-47 weeks after baseline). Among these, probable depression (risk ratio [RR], 1.32; 95% CI = 1.12-1.55), probable anxiety (RR = 1.42; 95% CI, 1.23-1.65), worry about COVID-19 (RR, 1.37; 95% CI, 1.17-1.61), perceived stress (highest vs lowest quartile: RR, 1.46; 95% CI, 1.18-1.81), and loneliness (RR, 1.32; 95% CI, 1.08-1.61) were each associated with post–COVID-19 conditions (1403 cases) in generalized estimating equation models adjusted for sociodemographic factors, health behaviors, and comorbidities. Participants with 2 or more types of distress prior to infection were at nearly 50% increased risk for post–COVID-19 conditions (RR, 1.49; 95% CI, 1.23-1.80). All types of distress were associated with increased risk of daily life impairment (783 cases) among individuals with post–COVID-19 conditions (RR range, 1.15-1.51).

**Conclusions and Relevance:**

The findings of this study suggest that preinfection psychological distress may be a risk factor for post–COVID-19 conditions in individuals with SARS-CoV-2 infection. Future work should examine the biobehavioral mechanism linking psychological distress with persistent postinfection symptoms.

## Key Points

**Question:**

Is psychological distress before SARS-CoV-2 infection associated with risk of COVID-19–related symptoms lasting 4 weeks or longer, known as post–COVID-19 conditions?

**Findings:**

This cohort study found that among participants who did not report SARS-CoV-2 infection at baseline (April 2020) and reported a positive SARS-CoV-2 test result over 1 year of follow-up (N = 3193), depression, anxiety, perceived stress, loneliness, and worry about COVID-19 were prospectively associated with a 1.3- to 1.5-fold increased risk of self-reported post–COVID-19 conditions, as well as increased risk of daily life impairment related to post–COVID-19 conditions.

**Meaning:**

In this study, preinfection psychological distress was associated with risk of post–COVID-19 conditions and daily life impairment in those with post–COVID-19 conditions.

## Teaser

This cohort study analyzes self-reported data from 3 large ongoing studies to determine whether high levels of psychological distress before SARS-CoV-2 infection are associated with increased risk of developing post–COVID-19 conditions, sometimes called long COVID.

## Introduction

Postacute sequelae of SARS-CoV-2, sometimes called long COVID, are defined as signs and symptoms consistent with COVID-19 that extend beyond 4 weeks from onset of infection and constitute an emerging health issue.^1^ Common symptoms of post–COVID-19 conditions include fatigue, brain fog, dyspnea, digestive issues, loss of taste and smell, and depression,^2,3^ which may persist for months after initial infection.^4^ Post–COVID-19 conditions are associated with reduced quality of life and an inability to return to work.^5,6,7^ Systematic reviews, primarily of hospitalized patients, have estimated that 54% to 73% of patients may experience post–COVID-19 conditions.^8,9^ A study of health care claims of nearly 2 million persons with COVID-19 found that 23% reported long-term symptoms.^10^

Post–COVID-19 conditions remain poorly understood, with no definitive etiology, prevention, or treatment.^11^ Older age, obesity, female sex, hypertension, immunosuppressive conditions, asthma, and severe acute-phase disease have been linked with higher risk of post–COVID-19 conditions.^3,12,13,14^ Psychological distress is associated with greater severity and longer duration of acute respiratory tract infections.^15,16^ However, only 3 studies have examined psychological factors as potential risk factors of post–COVID-19 conditions.^12,17,18^ The single prospective study found preexisting anxiety or depression was associated with decreased likelihood of full recovery,^18^ aligning with the results of the retrospective studies.^12,17^ Sustained psychological distress may cause activation of the hypothalamic-pituitary-adrenal axis and subsequent immune dysregulation.^19,20,21,22,23^ Other common manifestations of distress, such as loneliness and perceived stress, which have increased during the pandemic,^24,25,26^ have been implicated in chronic disease and early mortality,^27,28^ but have not been investigated as possible risk factors for post–COVID-19 conditions.

In this study, we examined whether preinfection psychological distress early in the pandemic, including depression, anxiety, loneliness, perceived stress, and worry, were associated with increased likelihood of developing post–COVID-19 conditions among individuals subsequently infected with SARS-CoV-2. We examined the extent to which established risk factors for COVID-19 severity (eg, hypertension, asthma) and common adverse health consequences of distress (eg, smoking, obesity) might account for possible associations.^3,12,13^ Further, we investigated whether, in participants with post–COVID-19 conditions, higher distress was associated with having a greater number of symptoms and impairment in daily activity.

## Methods

The study was approved by the Partners HealthCare system institutional review board. Return of questionnaires implied informed consent. Results are reported in accordance with the Strengthening the Reporting of Observational Studies in Epidemiology (STROBE) reporting guideline.

### Study Design and Population

Participants were drawn from 3 large ongoing longitudinal studies: Nurses’ Health Study II (NHSII), Nurses’ Health Study 3 (NHS3), and the Growing Up Today Study (GUTS). The NHSII, established in 1989, enrolled 116 429 US female nurses aged 25 to 42 years and followed up biennially.^29^ The NHS3, established in 2010, has recruited more than 49 000 US and Canadian female nurses 18 years and older, with enrollment ongoing and follow-up every 6 months. Male nurses were included beginning in 2015. GUTS began in 1996 when NHSII participants enrolled their offspring ages 9 to 17 years (n = 27 793).

From April 2020 to May 2020, 105 662 participants who returned the most recent main questionnaire of each cohort were invited to complete an online COVID-19 questionnaire. A total of 58 612 invited participants (55%) responded to this questionnaire from April 2020 to September 1, 2020 (henceforth termed *baseline*). Respondents were administered monthly surveys thereafter. Participants actively working at a health care center (n = 23 053) were administered additional weekly questionnaires. In August 2020, the surveys changed to quarterly administration (eFigure 1 in the Supplement). The end of follow-up for the current analysis was November 3, 2021.

Among 58 612 participants who responded to the baseline questionnaire, we restricted analysis to 54 960 participants who did not report history of a positive test for SARS-CoV-2 at baseline and returned at least 1 follow-up questionnaire. During 19 months of follow-up, 3752 participants (6%) reported a positive result on a SARS-CoV-2 antibody, antigen, or polymerase chain reaction test and the date of that test. We further excluded 249 participants who did not return the final questionnaire, and 310 participants who did not answer the post–COVID-19 condition question, leaving 3193 participants for analysis (eFigure 2 in the Supplement).

### Measures

#### Types of Distress

Distress was measured at baseline. Frequency of depressive and anxiety symptoms in the past 2 weeks was assessed with the 4-item Patient Health Questionnaire (PHQ-4), which consists of a 2-item depression measure (PHQ-2) and a 2-item anxiety measure (2-item Generalized Anxiety Disorder scale [GAD-2]).^30^ Responses ranged from 0 (not at all) to 3 (nearly every day). Scores of 3 or higher on the PHQ-2 or GAD-2 indicated probable depression or probable anxiety.^30,31,32,33^ Worry about COVID-19 was assessed with the item, “How worried are you about COVID-19?” Response options were not at all, not very worried, somewhat worried, and very worried.^34^ The reference was not at all or not very worried.

Two additional types of distress were assessed only among participants who were not active health care workers. The 4-item Perceived Stress Scale (PSS-4) queries frequency of past-month feelings of stress (eg, “difficulties piling up so high that you could not overcome them”).^35,36^ Response options ranged from never to very often (0-4). The summed score was divided into quartiles for analysis.

The 3-item UCLA Loneliness Scale queried the frequency of feeling lack of companionship, left out, and isolated from others (hardly ever [1], some of the time [2], or often [3]).^37^ We divided the score into 3 levels for analysis: hardly ever lonely (3 points, reference), less than some of the time (4-5 points), and some of the time or often (≥6 points).

For each participant, we calculated the number of distress types experienced at a high level,^30,31,32,33,34,35,36,37^ including probable depression, probable anxiety, somewhat or very worried about COVID-19, the top quartile of perceived stress, and lonely some of the time or more often (coded as 0, 1, or 2 or more types of distress).

#### SARS-CoV-2 Infection, Post–COVID-19 Conditions, and Post–COVID-19 Impairment

Positive SARS-CoV-2 test results (antibody, antigen, or polymerase chain reaction) in the past 7, 30, and 90 days; COVID-19 symptoms; and hospitalization due to COVID-19 occurring since March 1, 2020, were self-reported on all questionnaires. Post–COVID-19 conditions were assessed on the final questionnaire, administered 336 days after baseline. Participants were asked, “Have you experienced any long-term COVID-19 symptoms (lasting for more than 4 weeks)?”^1^ If yes, participants were asked to endorse any COVID-19–related symptoms they experienced, including fatigue, shortness of breath or difficulty breathing, persistent cough, muscle/joint/chest pain, smell/taste problems, confusion/disorientation/brain fog, memory issues, depression/anxiety/changes in mood, headache, intermittent fever, heart palpitations, rash/blisters/welts, mouth or tongue ulcers, or other symptoms. Participants with self-reported post–COVID-19 conditions were asked (1) whether they were still experiencing symptoms and (2) how often the symptoms prevented them from carrying out daily activities (never vs occasionally, often, usually, or always).

Nine COVID-19–related symptoms were also queried on each follow-up questionnaire (eMethods in the Supplement). Because a comprehensive list of post–COVID-19 symptoms was asked only on the final questionnaire, and because follow-up questionnaires were too infrequent to capture post–COVID-19 conditions for most participants, our primary analyses used the final report to ascertain case status.

#### Covariates

Covariates were queried before the pandemic. Date of birth, race and ethnicity (American Indian/Alaska Native, Asian, Black or African American, Native Hawaiian or Pacific Islander, White, or other), sex, and height were self-reported at cohort entry. We used the most recent report (NHS2: 2017; NHS3: 2010-2019; GUTS: 2018) of weight, smoking status (never, former, current), marital status (married or unmarried), and history of physician-diagnosed diabetes, hypertension, high cholesterol, asthma, and cancer. Body mass index (BMI) was calculated as weight in kilograms divided by height in meters squared. Socioeconomic status was measured using educational attainment of the participant (GUTS) or their spouse or partner (NHS2, NHS3). Health care worker status was queried at baseline.

### Statistical Analysis

Among participants who reported a positive SARS-CoV-2 test over follow-up, we compared participants who responded to the post–COVID-19 condition question with those who did not, regarding sociodemographic factors, COVID-19 severity risk factors, and types of distress at baseline. We then compared the prevalence of demographic factors and COVID-19 risk factors by level of distress at baseline.

We estimated relative risks (RRs) and 95% CIs for post–COVID-19 conditions using a general estimating equation (GEE) with a log link and Poisson distribution, with each type of distress or number of types of distress as the independent variable in separate models, adjusted for age, sex, racial identity, educational attainment, and health care worker status. To examine whether health-related factors in persons with vs without distress accounted for possible higher risk of post–COVID-19 conditions, we further adjusted for BMI and smoking status and additionally adjusted for comorbidities, including history of hypertension, diabetes, high cholesterol, asthma, and cancer. Furthermore, among participants with post–COVID-19 conditions, we compared prevalence of COVID-19 symptoms by distress at baseline and fit GEE models to examine the association between distress at baseline and any post–COVID-19 related daily life impairment. Missingness of each variable was less than 5%. Indicator variables were used for missing categorical variables. The median response was imputed for missing continuous variables.

We conducted 10 sensitivity analyses. First, to distinguish post–COVID-19 symptoms from symptoms related to distress, we excluded participants reporting only psychological, cognitive, or neurological symptoms. Second, to ensure that COVID-19–related symptoms did not precede infection, we excluded 846 participants reporting any COVID-19–related symptoms at baseline. Third, we considered as cases only participants reporting post–COVID-19 conditions both in follow-up questionnaires and in the final questionnaire (n = 1013). Fourth, to examine associations in men, we restricted analyses to 115 male participants reporting a positive test. Fifth, to reduce possible recall bias, we restricted post–COVID-19 condition cases to participants who reported ongoing symptoms (n = 1023). Sixth, we included 1584 participants who reported COVID-19 during follow-up but did not have a positive test. Seventh, as preinfection distress has been associated with severity of acute disease,^38^ we excluded 132 participants (4.1%) who were hospitalized because of COVID-19. Eighth, we multiply imputed post–COVID-19 condition status for 559 participants missing these data.^39^ Ninth, to ensure that symptoms at baseline did not affect distress measures, we excluded 69 individuals reporting infection within 4 weeks of baseline. Tenth, we defined post–COVID-19 conditions as cases having symptoms longer than 8 weeks. All analyses were conducted in SAS version 9.4 (SAS Institute). A 2-sided *P* < .05 was considered statistically significant.

## Results

The 54 960 participants were 96.5% White (n = 53 047), 96.6% female (n = 53 107), and 38.0% active health care workers (n = 20 902), with a mean (SD) age of 57.5 (13.8) years. Participants missing data about post–COVID-19 conditions, vs those nonmissing, were younger and more likely to be health care workers, be unpartnered, and have higher distress at baseline (eTable 1 in the Supplement). We documented 3193 participants with a positive SARS-CoV-2 test result over 19 months of follow-up. Median time from return of baseline questionnaire to positive SARS-CoV-2 test results was 30 weeks (range, 1-47 weeks). The mean (SD) age of these participants was 55.3 (13.8) years; 96.8% were White (n = 3091), 96.4% were female (n = 3078), and 49.0% were active health care workers (n = 1565).

At baseline, those who had probable depression or anxiety were younger, were more likely to be active health care workers, and had a higher prevalence of asthma (Table 1). Participants very worried about COVID-19 were more likely to be female, more likely to be from racial and ethnic minority groups, and more likely to have asthma than the reference group. Compared with participants who had the least perceived stress, those in the highest quartile were younger and more likely to have asthma. Participants who had probable depression, had probable anxiety, were very worried about COVID-19, were lonely, or were in the highest quartile of perceived stress were more likely to have a BMI of 35 or greater and to be unpartnered. All types of distress were correlated, with the strongest being depression and anxiety (Spearman ρ range, 0.14-0.63) (eTable 2 in the Supplement).

**Table 1.** Health and Demographic Characteristics by Types of Distress at Baseline (April-August 2020), Among Participants Who Tested Positive for SARS-CoV-2 During Follow-up, April 2020 Through November 2021 (N = 3193)^a^

Characteristic	No. (%)									
	Depressive symptoms		Anxiety symptoms		Worry about COVID-19		Perceived stress		Loneliness	
	No symptoms (n = 1595)	Probable depression (n = 397)	No symptoms (n = 1225)	Probable anxiety (n = 651)	No (n = 1000)	High (n = 496)	Lowest quartile (n = 514)	Highest quartile (n = 358)	Hardly ever (n = 467)	Some of the time or often (n = 456)
Age, mean (SD), y	57.4 (12.9)	50.8 (14.4)	59.5 (12.0)	50.5 (14.3)	56.3 (13.7)	55.5 (13.7)	63.0 (11.1)	51.1 (16.3)	60.3 (13.0)	56.7 (15.2)
Race, White^b^	1548 (97.1)	384 (96.7)	1185 (96.7)	634 (97.4)	978 (97.8)	473 (95.4)	505 (98.3)	349 (97.5)	454 (97.2)	444 (97.4)
Sex										
Female	1536 (96.3)	381 (96.0)	1179 (96.2)	628 (96.5)	948 (94.8)	486 (98.0)	492 (95.7)	337 (94.1)	446 (95.5)	430 (94.3)
Male	59 (3.7)	16 (4.0)	46 (3.8)	23 (3.5)	52 (5.2)	10 (2.0)	22 (4.3)	21 (5.9)	21 (4.5)	26 (5.7)
Active health care worker	753 (47.2)	224 (56.4)	522 (42.6)	361 (55.5)	436 (43.6)	236 (47.6)	NA	NA	NA	NA
Married	1194 (74.9)	237 (59.7)	926 (75.6)	422 (64.8)	724 (72.4)	312 (62.9)	399 (77.6)	223 (62.3)	361 (77.3)	296 (64.9)
Partner’s education high school or less^c^	209 (13.1)	38 (9.6)	186 (15.2)	58 (8.9)	124 (12.4)	53 (10.7)	68 (13.2)	29 (8.1)	58 (12.4)	45 (9.9)
BMI^d^										
25 to <30	474 (29.7)	104 (26.2)	378 (30.9)	180 (27.7)	310 (31.0)	150 (30.2)	169 (32.9)	88 (24.6)	149 (31.9)	132 (29.0)
30 to <35	310 (19.4)	76 (19.1)	256 (20.9)	112 (17.2)	179 (17.9)	79 (15.9)	102 (19.8)	58 (16.2)	86 (18.4)	86 (18.9)
≥35	202 (12.7)	93 (23.4)	158 (12.9)	128 (19.7)	118 (11.8)	113 (22.8)	73 (14.2)	71 (19.8)	62 (13.3)	75 (16.5)
Current smoker	36 (2.3)	15 (3.8)	34 (2.8)	20 (3.1)	25 (2.5)	17 (3.4)	11 (2.1)	8 (2.2)	15 (3.2)	14 (3.1)
Diabetes, ever	87 (5.5)	21 (5.3)	78 (6.4)	33 (5.1)	51 (5.1)	30 (6.1)	39 (7.6)	18 (5.0)	28 (6.0)	30 (6.6)
Hypertension, ever	320 (20.1)	89 (22.4)	274 (22.4)	147 (22.6)	209 (20.9)	127 (25.6)	137 (26.7)	80 (22.4)	113 (24.2)	121 (26.5)
High cholesterol, ever	395 (24.8)	105 (26.5)	326 (26.6)	170 (26.1)	253 (25.3)	143 (28.8)	136 (26.5)	76 (21.2)	111 (23.8)	136 (29.8)
Asthma, ever	180 (11.3)	78 (19.7)	120 (9.8)	111 (17.1)	109 (10.9)	79 (15.9)	40 (7.8)	61 (17.0)	50 (10.7)	60 (13.2)
Cancer, ever	80 (5.0)	19 (4.8)	60 (4.9)	29 (4.5)	45 (4.5)	28 (5.7)	32 (6.2)	25 (7.0)	24 (5.1)	26 (5.7)

Abbreviations: BMI, body mass index; NA, not applicable.

^a^ Values do not add to 100% because midlevels of variables, eg, subclinical depressive and anxious symptoms, somewhat worried, and less than some of the time of loneliness, are not shown because of space constraints; perceived stress and loneliness were queried only in participants who were not active health care workers (n = 1628).

^b^ Race and ethnicity were self-reported on cohort entry. Values for categories other than White (American Indian/Alaska Native, Asian, Black or African American, Native Hawaiian or Pacific Islander, and other) are not reported because their numbers were small.

^c^ Among participants who were married or in a domestic relationship among Nurses’ Health Study II and Nurses’ Health Study 3 participants. Participants’ own education attainment is listed for participants in the Growing Up Today Study.

^d^ Calculated as weight in kilograms divided by height in meters squared.

Of participants who reported a positive SARS-CoV-2 test result during follow-up, 43.9% (n = 1403, including 35 men) reported post–COVID-19 conditions. Among these, 86.9% (n = 1219) reported symptoms lasting 2 months or longer, and 55.8% (n = 783) reported at least occasional daily life impairment related to post–COVID-19 conditions. The most common symptoms were fatigue (56.0%, n = 786), smell or taste problems (44.6%, n = 626), shortness of breath (25.5%, n = 358), confusion/disorientation/brain fog (24.5%, n = 343), and memory issues (21.8%, n = 306) (eTable 3 in the Supplement). Agreement between measures of post–COVID-19 conditions using monthly/quarterly questionnaires and the final questionnaire was moderate (eTables 4-6 in the Supplement).

**Figure 1.**
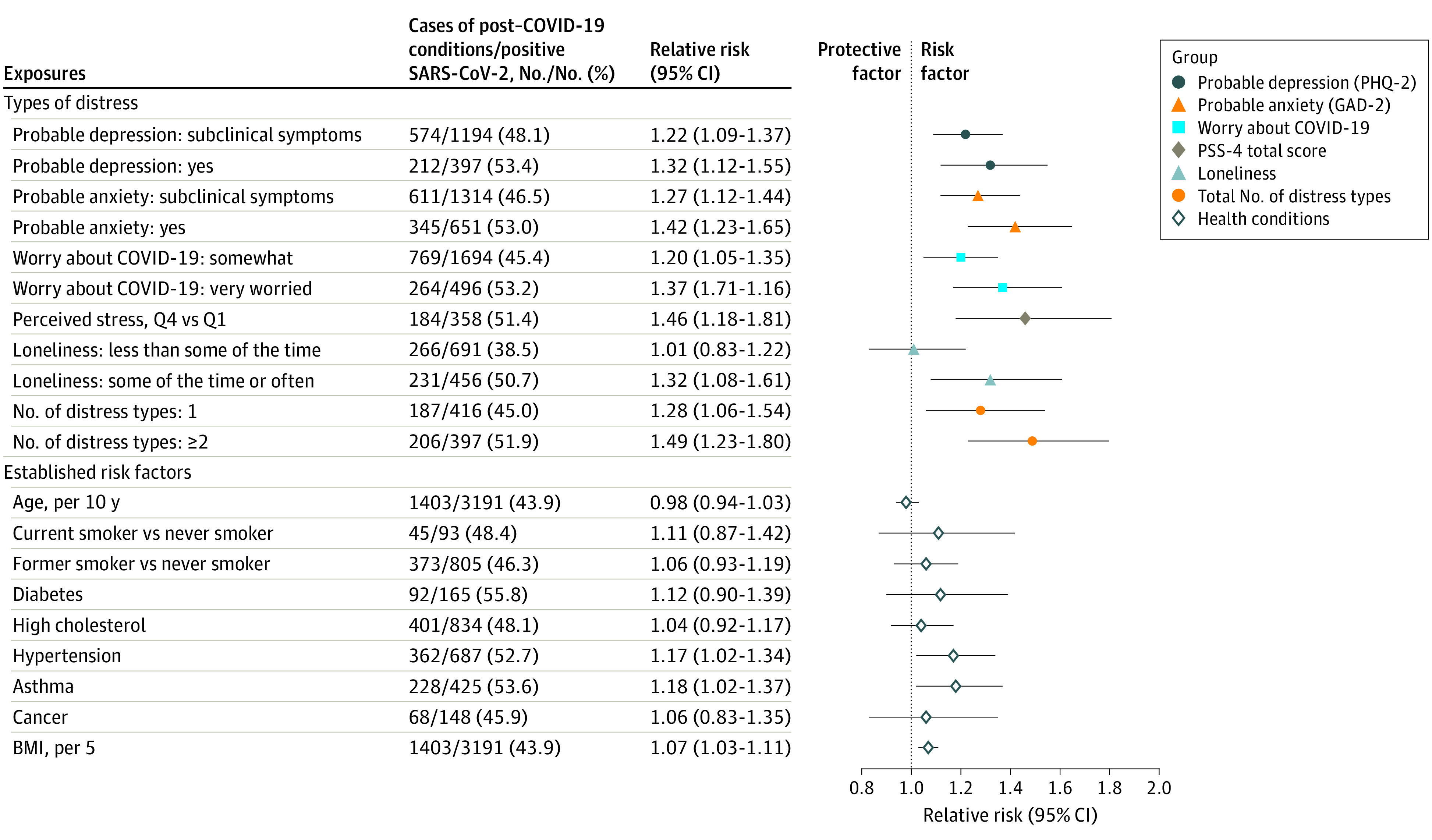
Association Between Distress and Established COVID-19 Risk Factors With Subsequent Post–COVID-19 Conditions, April 2020 Through November 2021 (N = 3193) All risk ratios are adjusted for covariates and established COVID-19 risk factors. Each type of distress is modeled separately as the independent variable, adjusted for age, sex, racial identity, health care worker status, partner’s education, body mass index (BMI), smoking status, and history of diabetes, hypertension, high cholesterol, asthma, and cancer. Estimates for established risk factors are from a single model with the following independent variables: age, sex, racial identity, health care worker status, partner’s education, BMI, smoking status, and history of diabetes, hypertension, high cholesterol, asthma, and cancer. Perceived stress and loneliness data were queried only in participants who were not active health care workers (n = 1628). Number of distress types is a count of probable depression, probable anxiety, somewhat or very worried about COVID, highest-quartile perceived stress, and lonely some of the time or often. BMI is calculated as weight in kilograms divided by height in meters squared. GAD-2 indicates 2-item Generalized Anxiety Disorder scale; PHQ-2, 2-item Patient Health Questionnaire; PSS-4, 4-item Perceived Stress Scale; Q, quartile.

All types of distress were significantly associated with increased risk of post–COVID-19 conditions in a dose-dependent manner after adjustment for demographic factors (probable depression, RR, 1.39 [95% CI, 1.19-1.63]; probable anxiety, RR, 1.47 [95% CI, 1.27-1.70]; very worried about COVID-19, RR, 1.43 [95% CI, 1.22-1.68]; highest quartile of perceived stress, RR, 1.50 [95% CI, 1.21-1.86]; lonely some of the time or often, RR, 1.35 [95% CI, 1.11-1.65]; all *P* < .01 for trend) (Table 2, model 1). Participants with more types of distress were at higher risk of developing post–COVID-19 conditions (≥2 types vs none, RR, 1.54; 95% CI, 1.28-1.86). In models further adjusted for smoking and BMI, associations between distress and post–COVID-19 conditions were attenuated by 1% to 4%. In models fully adjusted for comorbidities, all types of distress remained significantly associated with post–COVID-19 conditions (Table 2, models 2 and 3), and these associations were stronger than those with established risk factors (Figure 1).

**Table 2.** Association of Types of Distress and Risk of Subsequent Post–COVID-19 Conditions Among Persons With a Positive SARS-CoV-2 Test During Follow-up, April 2020 Through November 2021 (N = 3193)

Type of distress at study baseline	Risk ratio (95% CI)			
	Cases of post–COVID-19 conditions/positive SARS-CoV-2, No./No.	Model 1: Age, sex, racial identity, health care worker status, and partner’s education	Model 2: Model 1 further adjusted for smoking and BMI	Model 3: Model 2 further adjusted for comorbidities^a^
Probable depression (PHQ-2)				
No	614/1595	1 [Reference]	1 [Reference]	1 [Reference]
Subclinical symptoms	574/1194	1.25 (1.12-1.40)	1.23 (1.10-1.38)	1.22 (1.09-1.37)
Yes	212/397	1.39 (1.19-1.63)	1.34 (1.14-1.57)	1.32 (1.12-1.55)
*P* for trend^b^		<.001	<.001	<.001
Probable anxiety (GAD-2)				
No	447/1225	1 [Reference]	1 [Reference]	1 [Reference]
Subclinical symptoms	611/1314	1.28 (1.13-1.45)	1.28 (1.13-1.45)	1.27 (1.12-1.44)
Yes	345/651	1.47 (1.27-1.70)	1.44 (1.24-1.67)	1.42 (1.23-1.65)
*P* for trend^b^		<.001	<.001	<.001
Worry about COVID-19				
Not at all/not very worried	368/1000	1 [Reference]	1 [Reference]	1 [Reference]
Somewhat worried	769/1694	1.22 (1.07-1.38)	1.20 (1.06-1.36)	1.20 (1.05-1.35)
Very worried	264/496	1.43 (1.22-1.68)	1.38 (1.18-1.61)	1.37 (1.17-1.61)
*P* for trend^b^		<.001	<.001	<.001
Perceived stress^c^				
Quartile 1, 0-2 points (least)	188/514	1 [Reference]	1 [Reference]	1 [Reference]
Quartile 2, 3-4 points	166/432	1.07 (0.87-1.32)	1.07 (0.87-1.32)	1.07 (0.87-1.32)
Quartile 3, 5-6 points	135/315	1.21 (0.97-1.52)	1.22 (0.97-1.52)	1.19 (0.95-1.50)
Quartile 4, 7-14 points (most)	184/358	1.50 (1.21-1.86)	1.47 (1.19-1.82)	1.46 (1.18-1.81)
*P* for trend^b^		<.001	<.001	<.001
Loneliness^c^				
Hardly ever	177/467	1 [Reference]	1 [Reference]	1 [Reference]
Less than some of the time	266/691	1.02 (0.84-1.23)	1.01 (0.84-1.23)	1.01 (0.83-1.22)
Some of the time or often	231/456	1.35 (1.11-1.65)	1.34 (1.10-1.63)	1.32 (1.08-1.61)
*P* for trend^b^		.003	.004	.006
No. of types of distress^d^				
0	275/787	1 [Reference]	1 [Reference]	1 [Reference]
1	187/416	1.29 (1.07-1.55)	1.28 (1.06-1.54)	1.28 (1.06-1.54)
2 or more	206/397	1.54 (1.28-1.86)	1.50 (1.25-1.81)	1.49 (1.23-1.80)
*P* for trend^b^		<.001	<.001	<.001

Abbreviations: BMI, body mass index; GAD-2, 2-item Generalized Anxiety Disorder; PHQ-2, 2-item Patient Health Questionnaire.

^a^ Comorbidities included diabetes, hypertension, high cholesterol, asthma, and cancer.

^b^ *P* trend analysis used indicator levels as a continuous variable.

^c^ As perceived stress and loneliness were queried only in participants who were not active health care workers (n = 1628), risk ratios for number of types of distress were calculated only for these participants.

^d^ Number of types of distress is a count of probable depression, probable anxiety, somewhat or very worried about COVID-19, highest-quartile perceived stress, lonely some of the time or often.

Results were comparable in sensitivity analyses excluding cases of post–COVID-19 conditions with only psychological, cognitive, or neurological symptoms; excluding participants who reported any COVID-19–related symptoms at baseline; defining cases as individuals with reports of post–COVID-19 conditions in both follow-up and final questionnaires; restricted to male participants (RRs = 1.85-3.23); restricting cases to participants with ongoing symptoms at the final survey; excluding those hospitalized because of COVID-19; including presumed COVID-19 cases; using multiple imputation; and excluding cases with an onset within 4 weeks of baseline (eTable 7 in the Supplement). Results were somewhat stronger when we defined post–COVID-19 conditions as having symptoms longer than 8 weeks (eTable 8 in the Supplement).

**Figure 2.**
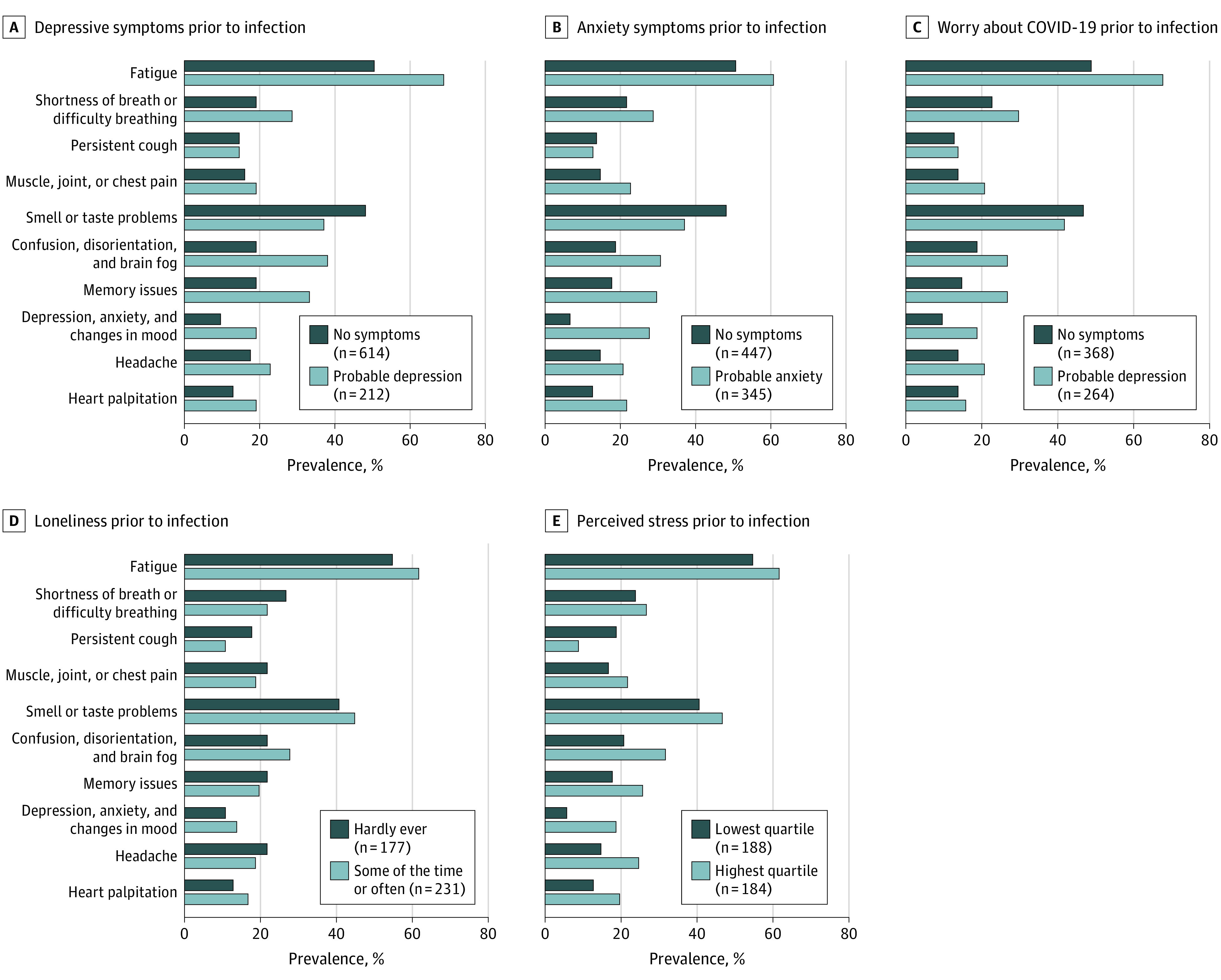
Prevalence of Long-term COVID-19–Related Symptoms by Types of Distress at Baseline (April-August 2020), Among 1403 Individuals With Post–COVID-19 Conditions Rare symptoms of post–COVID-19 conditions that are not presented because of space constraints include intermittent fever; rash, blisters, or welts anywhere on the body; and mouth or tongue ulcers. Values do not add to 100% because midlevels of variables (ie, subclinical depressive and anxious symptoms, somewhat worried, and loneliness less than some of the time) are not shown because of space constraints. Perceived stress and loneliness were queried only in participants who were not active health care workers.

**Figure 3.**
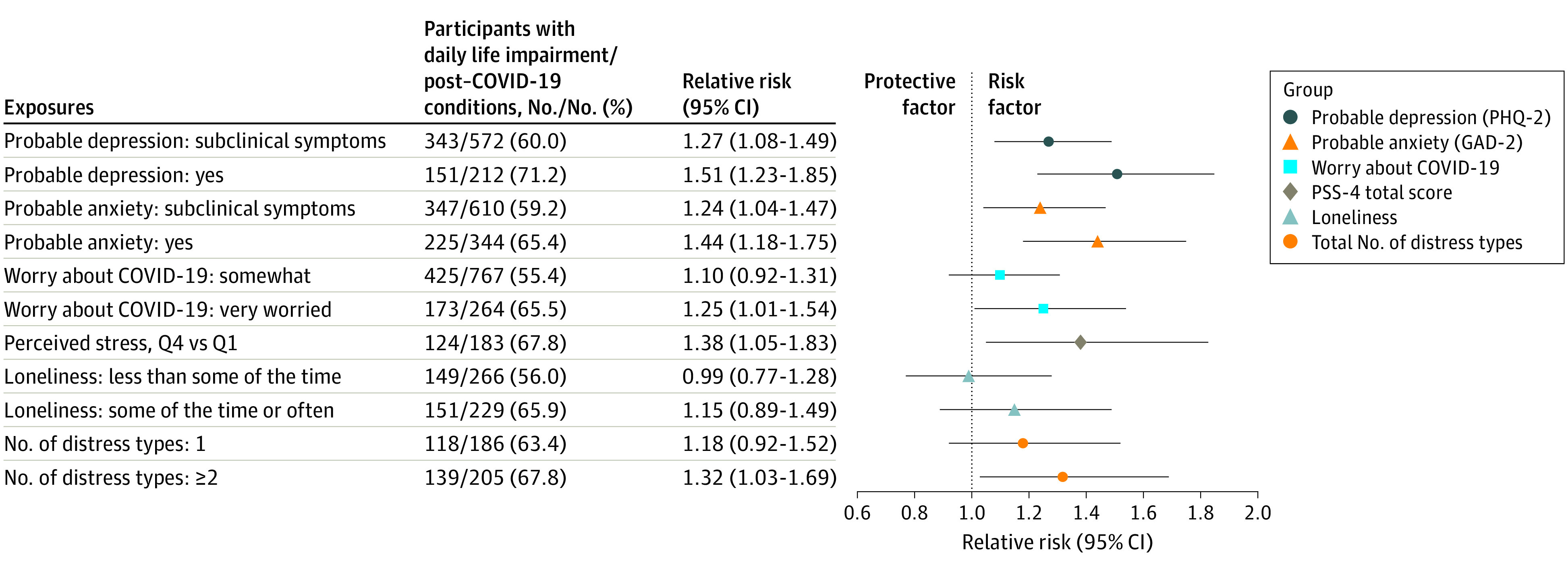
Association of Distress With Risk of Daily Life Impairment From Post–COVID-19 Conditions Among 1403 Individuals With Post–COVID-19 Conditions Each type of distress is modeled separately as the independent variable, adjusted for age, sex, racial identity, health care worker status, partner’s education, BMI, smoking status, and history of diabetes, hypertension, high cholesterol, asthma, and cancer. Perceived stress and loneliness were queried only in participants who were not active health care workers. Number of types of distress is a count of probable depression, probable anxiety, somewhat or very worried about COVID-19, highest-quartile perceived stress, and lonely some of the time or often. GAD-2 indicates 2-item Generalized Anxiety Disorder; PHQ-2, 2-item Patient Health Questionnaire; PSS-4, 4-item Perceived Stress Scale; Q, quartile.

Furthermore, among participants reporting post–COVID-19 conditions, we examined the association of preinfection distress with symptoms and impairment. All COVID-19 symptoms, except for persistent cough and smell or taste problems, were more prevalent in participants with vs without each type of distress (Figure 2). Individuals with distress at baseline reported a greater number of symptoms of post–COVID-19 condition (eg, probable depression, mean [SD] symptoms = 3.4 [2.1]; no depression, mean [SD] symptoms = 2.5 [1.7]). Symptoms of depression, symptoms of anxiety, worry, and perceived stress at baseline were associated with a 25% to 51% increased risk of having symptoms that interfered with activities occasionally to always (Figure 3).

## Discussion

In this prospective study of individuals followed up for more a year starting in April 2020, we found preinfection psychological distress, including symptoms of depression, symptoms of anxiety, worry about COVID-19, loneliness, and perceived stress, was strongly associated with risk of post–COVID-19 conditions among those infected with SARS-CoV-2. These associations remained significant after further adjustment for health-related factors. We found a dose-dependent association between number of types of distress and risk of post–COVID-19 conditions, with participants who experienced high levels of 2 or more types of distress at nearly 50% greater risk of post–COVID-19 conditions than those who did not experience a high level of distress. Participants with vs without distress at baseline developed on average 1 additional long-term symptom. All types of distress except loneliness were associated with risk of daily life impairment related to post–COVID-19 conditions.

Few studies have examined distress as a risk factor for post–COVID-19 conditions. A prospective study using a UK cohort of more than 7000 patients with multiple sclerosis found preexisting anxiety and depression were associated with 29% decreased risk of full recovery at median follow-up of 87 days.^18^ Distress at the time of infection has been associated with longer and more severe upper respiratory tract infections.^15,40^ Prior studies have further suggested that distress is associated with long-term symptoms after Lyme infection and in functional syndromes, eg, chronic fatigue syndrome and fibromyalgia, that have symptoms similar to those of post–COVID-19 conditions, such as fatigue, headache, and muscle pain.^41,42,43,44,45,46^

Our results should not be misinterpreted as supporting a hypothesis that post–COVID-19 conditions are psychosomatic.^47^ First, among respondents who developed post–COVID-19 conditions, more than 40% had no distress at baseline. Second, symptoms of post–COVID-19 conditions differ substantially from symptoms of mental illness. Although fatigue and brain fog may occur with depression, smell and taste problems, shortness of breath and difficulty breathing, and cough are not common symptoms of mental illness.^48^ Third, more than 50% of patients with post–COVID-19 conditions report relapses triggered by physical activity.^49^ In contrast, physical activity is protective against relapse of mental illness.^48,50^ Fourth, results were similar when excluding participants reporting only psychiatric, cognitive, or neurological symptoms.

Inflammation and immune dysregulation may link psychological distress with post–COVID-19 conditions. Distress is associated with chronic systemic inflammation, resulting in sustained production of proinflammatory cytokines and reactive oxygen species.^19,20,21,22,23^ Inflammatory cytokines have been proposed as possible causes of respiratory, neurological, cardiovascular, muscular, and gastrointestinal long-term COVID-19 symptoms.^51,52,53^ In addition, stress activates the hypothalamic-pituitary-adrenal axis, which can lead to chronic immune suppression. Immunosuppressive conditions have been found to be associated with risk of persistent symptoms after COVID-19,^12^ but findings were not conclusive.^13,54,55^ Furthermore, autoantibodies have been associated with both mental health conditions and post–COVID-19 conditions.^14,56^ In the central nervous system, mental health disorders are associated with chronic low-grade inflammation and microglia activation, which may cause cognitive impairment and long-term fatigue.^57^ Hypometabolism in the frontal lobe and cerebellum, a pathopsychological change associated with major depression, has also been implicated in post–COVID-19 fatigue.^58,59,60^

### Limitations and Strengths

Our study has several limitations. First, our study population was predominantly White and female and had a significant proportion of health care personnel, limiting generalizability. Second, a positive result on a SARS-CoV-2 test was self-reported, though self-reported health information has had high validity in these cohorts.^61,62^ Third, data were not missing at random, which might have introduced bias. However, results were similar in analyses using multiple imputation. Fourth, the agreement between symptoms reported on the final and monthly/quarterly questionnaires was moderate, suggesting a chance of outcome misclassification, which may bias our results toward null. Nevertheless, symptoms of post–COVID-19 conditions change over the course of the illness.^63^ Fifth, depression and anxiety were measured using validated scales rather than clinical diagnoses.

Our study has several strengths. Monthly/quarterly surveys were sent to 3 large cohorts prospectively measuring incident infection and COVID-19 symptoms during an active stage of the pandemic. Distress was measured early in the pandemic, which may have more accurately captured recent distress, compared with studies using prepandemic medical records. We examined some common yet largely unstudied types of distress, including loneliness, perceived stress, and worry about COVID-19.

## Conclusions

The findings of this study suggest that preexisting psychological distress is associated with subsequent risk of developing post–COVID-19 conditions. Further research should investigate whether interventions that reduce distress help prevent or treat post–COVID-19 conditions. Identification and treatment of biological pathways linking distress with long-term COVID-19 symptoms may benefit individuals with post–COVID-19 conditions or other chronic postinfection syndromes.

## Notes

Supplement. **eFigure 1.** Questionnaire sequence for the COVID-19 study, April 2020 to November 2021 **eFigure 2.** Flow chart of study design and study population selection, April 2020 to November 2021
eMethods
**eTable 1.** Health and demographic characteristics at baseline (April-September 2020) by missing long COVID status, among participants who ever tested positive for SARS-CoV-2 between April 2020 - November 2021, N=3,752 **eTable 2.** Spearman correlations between types of distress among persons with a positive SARS-CoV-2 test from April 2020 to November 2021, NHS II, NHS3, and GUTS, N=3,193 **eTable 3.** Prevalence of long-COVID-related symptoms among participants with long COVID, N=1,403 **eTable 4.** Long COVID status ascertained on monthly or quarterly versus final questionnaire among 3,193 participants infected with SARS-COV-2 **eTable 5.** Prevalence of COVID-19 related symptoms reported on monthly or quarterly follow up and the final questionnaire among persons who had eligible measures on both questionnaires, N=1,381 **eTable 6.** Agreement between COVID-19 related symptoms reported on monthly or quarterly versus final questionnaires, among participants data on both types of questionnaires, N=1,381 **eTable 7.** Sensitivity analysis for the association of types of distress and subsequent risk of long COVID among persons with a positive SARS-CoV-2 test during follow-up, April 2020 - November 2021 **eTable 8.** Association of types of distress and risk of subsequent long COVID (more than 8 weeks) among persons with a positive SARS-CoV-2 test during follow-up, April 2020 - November 2021, N=3,142
