# Supplementary material for: Associations of Depression, Anxiety, Worry, Perceived Stress, and Loneliness Prior to Infection With Risk of Post–COVID-19 Conditions
Source: JAMA Psychiatry. 2022 Sep 7;79(11):1081–91. doi: 10.1001/jamapsychiatry.2022.2640 (PMC9453634; doi:10.1001/jamapsychiatry.2022.2640)
Supplement: Supplement. — eFigure 1. Questionnaire sequence for the COVID-19 study, April 2020 to November 2021 eFigure 2. Flow chart of study design and study population selection, April 2020 to November 2021 eMethods eTable 1. Health and demographic characteristics at baseline (April-September 2020) by missing long COVID status, among participants who ever tested positive for SARS-CoV-2 between April 2020 - November 2021, N=3,752 eTable 2. Spearman correlations between types of distress among persons with a positive SARS-CoV-2 test from April 2020 to November 2021, NHS II, NHS3, and GUTS, N=3,193 eTable 3. Prevalence of long-COVID-related symptoms among participants with long COVID, N=1,403 eTable 4. Long COVID status ascertained on monthly or quarterly versus final questionnaire among 3,193 participants infected with SARS-COV-2 eTable 5. Prevalence of COVID-19 related symptoms reported on monthly or quarterly follow up and the final questionnaire among persons who had eligible measures on both questionnaires, N=1,381 eTable 6. Agreement between COVID-19 related symptoms reported on monthly or quarterly versus final questionnaires, among participants data on both types of questionnaires, N=1,381 eTable 7. Sensitivity analysis for the association of types of distress and subsequent risk of long COVID among persons with a positive SARS-CoV-2 test during follow-up, April 2020 - November 2021 eTable 8. Association of types of distress and risk of subsequent long COVID (more than 8 weeks) among persons with a positive SARS-CoV-2 test during follow-up, April 2020 - November 2021, N=3,142 [file jamapsychiatry-e222640-s001.pdf]

Supplemental Online Content

Wang S, Quan L, Chavarro JE, et al. Associations of depression, anxiety, worry, perceived stress, and loneliness prior to infection with risk of post–COVID-19 conditions. *JAMA Psychiatry*. Published online September 7, 2022.
doi:10.1001/jamapsychiatry.2022.2640

eFigure 1. Questionnaire sequence for the COVID-19 study, April 2020 to November 2021

eFigure 2. Flow chart of study design and study population selection, April 2020 to November 2021

eMethods

eTable 1. Health and demographic characteristics at baseline (April-September 2020) by missing long COVID status, among participants who ever tested positive for SARS-CoV-2 between April 2020 - November 2021, N=3,752

eTable 2. Spearman correlations between types of distress among persons with a positive SARS-CoV-2 test from April 2020 to November 2021, NHS II, NHS3, and GUTS, N=3,193

eTable 3. Prevalence of long-COVID-related symptoms among participants with long COVID, N=1,403

eTable 4. Long COVID status ascertained on monthly or quarterly versus final questionnaire among 3,193 participants infected with SARS-COV-2

eTable 5. Prevalence of COVID-19 related symptoms reported on monthly or quarterly follow up and the final questionnaire among persons who had eligible measures on both questionnaires, N=1,381

eTable 6. Agreement between COVID-19 related symptoms reported on monthly or quarterly versus final questionnaires, among participants data on both types of questionnaires, N=1,381

eTable 7. Sensitivity analysis for the association of types of distress and subsequent risk of long COVID among persons with a positive SARS-CoV-2 test during follow-up, April 2020 - November 2021

eTable 8. Association of types of distress and risk of subsequent long COVID (more than 8 weeks) among persons with a positive SARS-CoV-2 test during follow-up, April 2020 - November 2021, N=3,142

This supplemental material has been provided by the authors to give readers additional information about their work.

© 2022 Wang S et al. *JAMA Psychiatry.*

eFigure 1. Questionnaire sequence for the COVID-19 study, April 2020 to November 2021

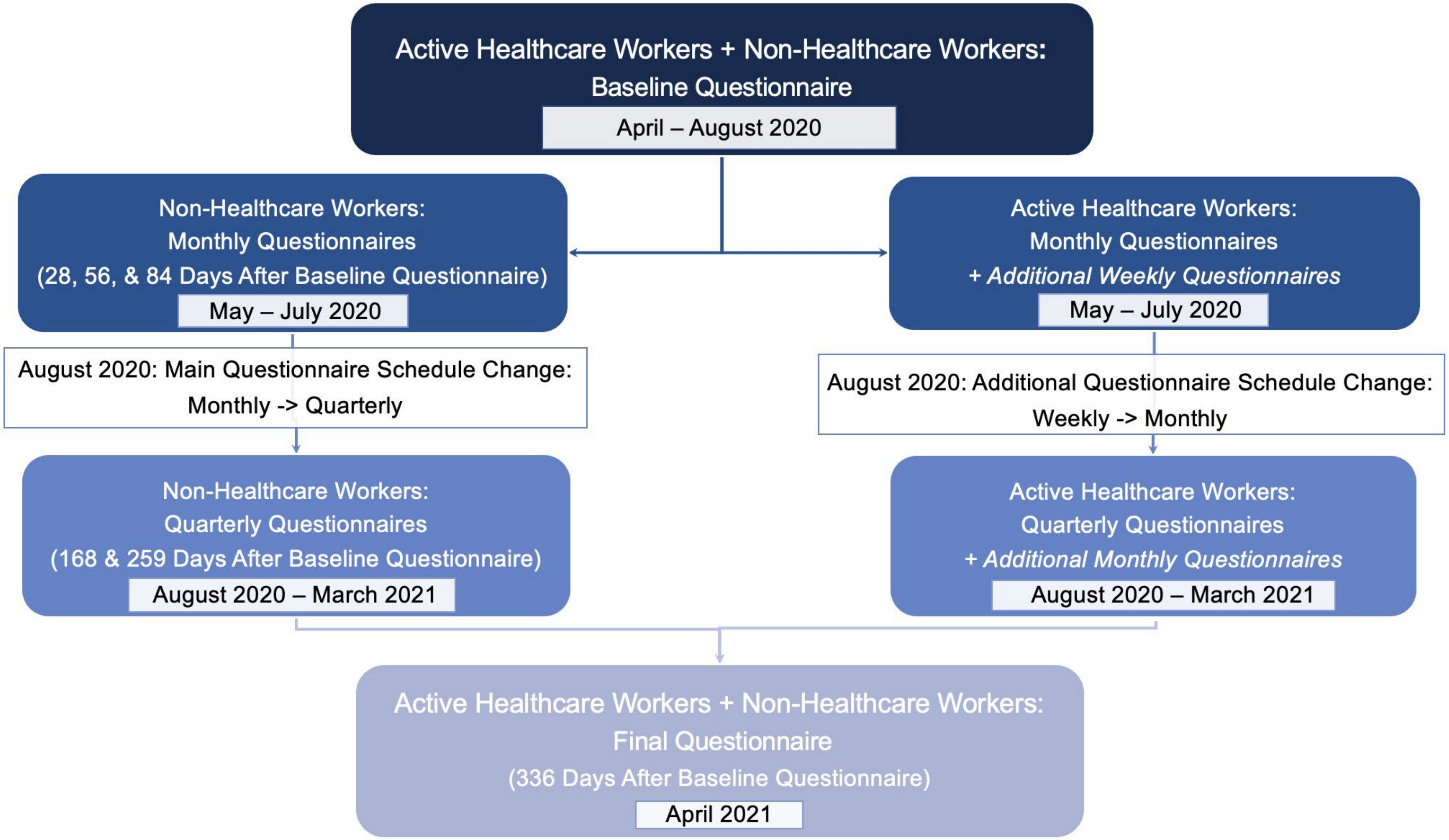

© 2022 Wang S et al. *JAMA Psychiatry.*

eFigure 2. Flow chart of study design and study population selection, April 2020 to November 2021

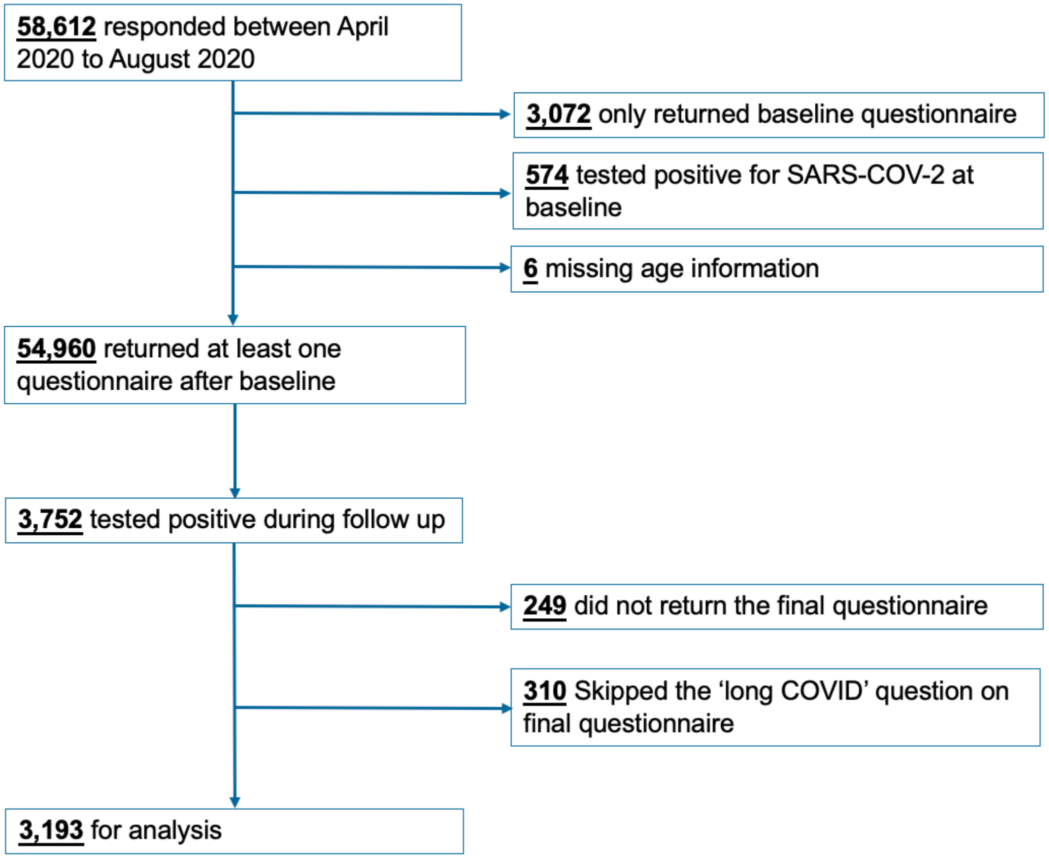

© 2022 Wang S et al. *JAMA Psychiatry.*

eMethods

Nine COVID-19-related symptoms were queried on each follow-up questionnaire, for each of three timeframes: past-7, -30, and -90 days. Participants were asked, "during the past [7/30/90] days, have you experienced any of the following symptoms: persistent cough, shortness of breath, fever, sore throat, muscle aches, digestive symptoms (vomiting, diarrhea), loss of taste, loss of smell, or other symptoms consistent with COVID-19 infection?" We identified participants who reported COVID-19 symptoms lasting ≥30 days, using the reported date of positive test as the beginning of symptoms, and conservatively calculating the date of last symptoms as: return date of the last questionnaire on which any symptom was reported - the timeframe (e.g., 7, 30, or 90 days). In a sensitivity analysis, we considered as cases only participants reporting long COVID both in follow-up questionnaires and in the final questionnaire.

© 2022 Wang S et al. *JAMA Psychiatry.*

eTable 1. Health and demographic characteristics at baseline (April-September 2020) by missing long COVID status, among participants who ever tested positive for SARS-CoV-2 between April 2020 - November 2021, N=3,752.

	No. (%)			
	Missing 'long COVID' status			
	Not missing	Missing		
	N=3193	Did not return final questionnaire or left blank on the final questionnaire N=559	Did not return final questionnaire (N=249)	Left blank on the final questionnaire (N=310)
Age, mean (SD), years	55.3 (13.8)	52.6 (14.2)	50.6 (14.8)	54.3 (13.4)
Race				
White	3091 (96.8)	538 (96.2)	245 (98.4)	293 (94.5)
Sex				
Female	3078 (96.4)	536 (95.9)	232 (93.2)	304 (98.1)
Active healthcare worker, yes	1565 (49.0)	368 (65.8)	144 (57.8)	224 (72.3)
Married, yes	2231 (69.9)	335 (59.9)	150 (60.2)	185 (59.7)
Partner's education[a]				
High school or lower	388 (12.2)	45 (8.1)	22 (8.8)	23 (7.4)
BMI, kg/m^2				
25 to <30	958 (30.0)	154 (27.6)	65 (26.1)	89 (28.7)
30 to <35	591 (18.5)	90 (16.1)	45 (18.1)	45 (14.5)
⩾35	488 (15.3)	63 (11.3)	34 (13.7)	29 (9.4)
Smoking status				
Current smoker	93 (2.9)	25 (4.5)	13 (5.2)	12 (3.9)
Diabetes, ever	166 (5.2)	24 (4.3)	15 (6.0)	9 (2.9)
Hypertension, ever	688 (21.6)	86 (15.4)	41 (16.5)	45 (14.5)
High cholesterol, ever	835 (26.2)	130 (23.3)	57 (22.9)	73 (23.6)
Asthma, ever	425 (13.3)	81 (14.5)	34 (13.7)	47 (15.2)
Cancer, ever	148 (4.6)	24 (4.3)	9 (3.6)	15 (4.8)
Probable depression, yes	397 (12.5)	86 (15.4)	39 (15.7)	47 (15.2)
Probable anxiety, yes	651 (20.4)	133 (23.9)	53 (21.5)	80 (25.9)
Very worried about COVID	496 (15.6)	109 (19.5)	35 (14.1)	74 (23.9)
Loneliness, some of the time or often	456 (28.3)	60 (31.9)	26 (25.2)	34 (40.0)
Perceived stress, mean (SD)	1.3 (1.1)	1.5 (1.1)	1.4 (1.1)	1.6 (1.1)

Note. Perceived stress and loneliness were collected only in persons who were not active health care workers.

© 2022 Wang S et al. *JAMA Psychiatry.*

eTable 2. Spearman correlations between types of distress among persons with a positive SARS-CoV-2 test from April 2020 to November 2021, NHS II, NHS3, and GUTS, N=3,193.

	Depressive symptoms	Anxiety symptoms	Worry about COVID	Perceived stress	Loneliness
Depressive symptoms	1.0	-	-	-	-
Anxiety symptoms	ρ=0.63[a]	1.0	-	-	-
Worry about COVID	ρ=0.23[a]	ρ=0.30[a]	1.0	-	-
Perceived stress	ρ=0.54[a]	ρ=0.54[a]	ρ=0.22[a]	1.0	-
Loneliness	ρ=0.46[a]	ρ=0.32[a]	ρ=0.14[a]	ρ=0.39[a]	1.0

Note. NHS = Nurses' Health Study; GUTS = Growing Up Today Study. Perceived stress and loneliness were queried in non-active health care workers only.

[a]*P*<.001

© 2022 Wang S et al. *JAMA Psychiatry.*

eTable 3. Prevalence of long-COVID-related symptoms among participants with long COVID, N=1,403

Symptoms	N	%
Fatigue	786	56.0
Smell or taste problems	626	44.6
Shortness of breath or difficulty breathing	358	25.5
Confusion, disorientation, 'brain fog'	343	24.5
Memory issues	306	21.8
Muscle, joint of chest pain	260	18.5
Headache	254	18.1
Heart palpitations	225	16.0
Depression, anxiety, changes in mood	208	14.8
Persistent cough	184	13.1
Intermittent fever	34	2.4
Mouth or tongue ulcers	34	2.4
Rash, blisters, or welts anywhere on body	32	2.3

© 2022 Wang S et al. *JAMA Psychiatry.*

eTable 4. Long COVID status ascertained on monthly or quarterly versus final questionnaire among 3,193 participants infected with SARS-COV-2.

		No. (%)		
		Monthly or quarterly questionnaires		
		Yes (N=950)	No (N=431)	Undetermined (N=1812)
Final questionnaire	Yes (N=1790)	560 (59.0%)	138 (32.0%)	1092 (60.3%)
	No (N=1403)	390 (41.1%)	293 (68.0%)	720 (39.7%)

Note. "Undetermined" participants were those who did not return monthly or quarterly questionnaires within a timeframe enabling ascertainment of long COVID.

© 2022 Wang S et al. *JAMA Psychiatry.*

eTable 5. Prevalence of COVID-19 related symptoms reported on monthly or quarterly follow up and the final questionnaire among persons who had eligible measures on both questionnaires, N=1,381.

	No. (%)	
	'Past 7- 30- or 90-day symptoms" reported on monthly or quarterly questionnaires	'When you had COVID': symptoms reported on final questionnaire
Symptoms, ever		
Persistent cough	471 (34.2)	530 (38.5)
Shortness of breath or difficulty breathing	345 (25.0)	423 (30.7)
Fever	406 (29.5)	652 (47.3)
Sore throat	539 (39.1)	411 (29.8)
Muscle aches	639 (46.4)	865 (62.7)
Digestive symptoms	488 (35.4)	312 (22.6)
Loss of taste	389 (28.2)	721 (52.3)
Loss of smell	411 (29.8)	754 (54.7)

© 2022 Wang S et al. *JAMA Psychiatry.*

eTable 6. Agreement between COVID-19 related symptoms reported on monthly or quarterly versus final questionnaires, among participants with data on both types of questionnaires, N=1,381.

	'Past 7- 30- or 90-day symptoms' reported on monthly or quarterly questionnaires							
	Persistent cough	Shortness of breath	Fever	Sore throat	Muscle pain	Digestive symptoms	Loss of taste	Loss of smell
	Agreement (Cohen's kappa)							
'When you had COVID': symptoms reported on final questionnaire								
Persistent cough	0.36							
Shortness of breath		0.49						
Fever			0.38					
Sore throat				0.32				
Muscle pain					0.30			
Digestive symptoms						0.30		
Loss of taste							0.41	
Loss of smell								0.42

© 2022 Wang S et al. *JAMA Psychiatry.*

eTable 7. Sensitivity analyses for the association of types of distress and subsequent risk of long COVID among persons with a positive SARS-CoV-2 test during follow-up, April 2020 - November 2021

	Main model	Model 1: Main model, excluding 304 long COVID cases with only psychiatric, cognitive, or neurological symptoms[a]	Model 2: Main model, excluding 846 participants with any COVID-19-related symptoms at baseline	Model 3: Main model, excluding 528 participants with discordant long COVID status	Model 4: Main model, restricting to 115 men
Probable depression (PHQ-2)					
No	1.0 [Reference]	1.0 [Reference]	1.0 [Reference]	1.0 [Reference]	1.0 [Reference]
Subclinical symptoms	**1.25 (1.12-1.40)**	**1.29 (1.13-1.47)**	**1.26 (1.10-1.45)**	**1.29 (1.13-1.48)**	1.94 (0.91-4.12)
Yes	**1.39 (1.19-1.63)**	**1.39 (1.15-1.66)**	**1.32 (1.07-1.63)**	**1.41 (1.16-1.71)**	2.19 (0.74-6.47)
P trend[c]	**<.001**	**<.001**	**<.001**	**<.001**	0.07
Probable anxiety (GAD-2)					
No	1.0 [Reference]	1.0 [Reference]	1.0 [Reference]	1.0 [Reference]	1.0 [Reference]
Subclinical symptoms	**1.28 (1.13-1.45)**	**1.32 (1.14-1.52)**	**1.29 (1.12-1.50)**	**1.32 (1.14-1.53)**	**2.54 (1.12-5.78)**
Yes	**1.47 (1.27-1.70)**	**1.55 (1.31-1.82)**	**1.46 (1.22-1.74)**	**1.54 (1.30-1.83)**	1.85 (0.66-5.15)
P trend[c]	**<.001**	**<.001**	**<.001**	**<.001**	0.12
Worry about COVID					
Not at all/not very worried	1.0 [Reference]	1.0 [Reference]	1.0 [Reference]	1.0 [Reference]	1.0 [Reference]
Somewhat worried	**1.22 (1.07-1.38)**	**1.22 (1.06-1.40)**	**1.28 (1.10-1.48)**	**1.32 (1.13-1.53)**	1.92 (0.87-4.26)
Very worried	**1.43 (1.22-1.68)**	**1.47 (1.23-1.76)**	**1.46 (1.20-1.78)**	**1.50 (1.24-1.82)**	**3.23 (1.07-9.79)**
P trend[c]	**<.001**	**<.001**	**<.001**	**<.001**	**.03**
Perceived stress					
Q1, 0-2 points, (least)	1.0 [Reference]	1.0 [Reference]	1.0 [Reference]	1.0 [Reference]	1.0 [Reference]
Q2, 3-4 points	1.07 (0.87-1.32)	1.06 (0.84-1.34)	1.07 (0.85-1.36)	1.02 (0.79-1.31)	1.18 (0.40-3.46)
Q3, 5-6 points	1.21 (0.97-1.52)	1.19 (0.92-1.53)	1.16 (0.89-1.52)	1.22 (0.93-1.59)	2.45 (0.72-8.29)
Q4, 7-14 points, (most)	**1.50 (1.21-1.86)**	**1.51 (1.19-1.92)**	**1.51 (1.17-1.95)**	**1.58 (1.22-2.03)**	2.46 (0.81-7.45)
P trend[c]	**<.001**	**<.001**	**<.001**	**<.001**	.06
Loneliness					
Hardly ever	1.0 [Reference]	1.0 [Reference]	1.0 [Reference]	1.0 [Reference]	1.0 [Reference]
Less than some of the time	1.02 (0.84-1.23)	0.99 (0.80-1.23)	1.02 (0.82-1.27)	1.02 (0.81-1.28)	3.04 (0.85-10.93)
Some of the time or often	**1.35 (1.11-1.65)**	**1.34 (1.07-1.67)**	**1.31 (1.04-1.66)**	**1.43 (1.13-1.81)**	3.15 (0.79-12.52)
P trend[c]	**.003**	**.01**	**.02**	**.003**	.13

© 2022 Wang S et al. *JAMA Psychiatry.*

(continued)

	Model 5: Main model, restricting cases to 1,023 participants with ongoing symptoms at final assessment	Model 6: Main model, including 1,584 presumed SARS-CoV-2 infections as cases	Model 7: Main model, excluding 132 participants hospitalized for COVID-19	Model 8: Main model, multiply imputing missing long COVID status for 559 participants[b]	Model 9: Main model, excluding 69 cases onsetting within 4 weeks of baseline
Probable depression (PHQ-2)					
No	1.0 [Reference]	1.0 [Reference]	1.0 [Reference]	1.0 [Reference]	1.0 [Reference]
Subclinical symptoms	**1.32 (1.15-1.51)**	**1.20 (1.09-1.33)**	**1.25 (1.11-1.41)**	**1.25 (1.12-1.39)**	**1.24 (1.11-1.40)**
Yes	**1.60 (1.34-1.91)**	**1.40 (1.23-1.60)**	**1.41 (1.20-1.67)**	**1.36 (1.17-1.58)**	**1.39 (1.19-1.63)**
P trend[c]	**<.001**	**<.001**	**<.001**	**<.001**	**<.001**
Probable anxiety (GAD-2)					
No	1.0 [Reference]	1.0 [Reference]	1.0 [Reference]	1.0 [Reference]	1.0 [Reference]
Subclinical symptoms	**1.29 (1.11-1.49)**	**1.21 (1.09-1.35)**	**1.29 (1.13-1.46)**	**1.28 (1.13-1.44)**	**1.29 (1.13-1.46)**
Yes	**1.57 (1.33-1.86)**	**1.42 (1.25-1.60)**	**1.50 (1.29-1.75)**	**1.46 (1.27-1.68)**	**1.48 (1.28-1.72)**
P trend[c]	**<.001**	**<.001**	**<.001**	**<.001**	**<.001**
Worry about COVID					
Not at all/not very worried	1.0 [Reference]	1.0 [Reference]	1.0 [Reference]	1.0 [Reference]	1.0 [Reference]
Somewhat worried	**1.23 (1.06-1.42)**	**1.26 (1.13-1.40)**	**1.21 (1.07-1.38)**	**1.22 (1.08-1.38)**	**1.21 (1.07-1.37)**
Very worried	**1.53 (1.27-1.83)**	**1.48 (1.30-1.70)**	**1.43 (1.21-1.69)**	**1.44 (1.24-1.67)**	**1.43 (1.22-1.68)**
P trend[c]	**<.001**	**<.001**	**<.001**	**<.001**	**<.001**
Perceived stress					
Q1, 0-2 points, (least)	1.0 [Reference]	1.0 [Reference]	1.0 [Reference]	1.0 [Reference]	1.0 [Reference]
Q2, 3-4 points	1.14 (0.88-1.46)	1.12 (0.94-1.34)	1.07 (0.86-1.34)	1.08 (0.88-1.33)	1.07 (0.87-1.32)
Q3, 5-6 points	1.26 (0.95.1.66)	**1.24 (1.03-1.49)**	1.20 (0.95-1.53)	1.22 (0.98-1.53)	1.20 (0.96-1.51)
Q4, 7-14 points, (most)	**1.82 (1.42-2.34)**	**1.50 (1.26-1.79)**	**1.51 (1.21-1.90)**	**1.47 (1.20-1.82)**	**1.50 (1.20-1.86)**
P trend[c]	**<.001**	**<.001**	**<.001**	**<.001**	**<.001**
Loneliness					
Hardly ever	1.0 [Reference]	1.0 [Reference]	1.0 [Reference]	1.0 [Reference]	1.0 [Reference]
Less than some of the time	0.99 (0.79-1.25)	0.98 (0.84-1.15)	0.99 (0.81-1.21)	1.03 (0.86-1.25)	1.02 (0.84-1.24)
Some of the time or often	**1.40 (1.11-1.77)**	**1.29 (1.09-1.51)**	**1.33 (1.08-1.64)**	**1.34 (1.11-1.63)**	**1.35 (1.10-1.65)**
P trend[c]	**.005**	**.002**	**.007**	**.002**	**.003**

© 2022 Wang S et al. *JAMA Psychiatry.*

Note. PHQ-2 = 2-item Patient Health Questionnaire-2; GAD-2 = 2-item Generalized Anxiety Disorder; perceived stress and loneliness were queried in non-active health care workers only; Q=quartile.

Models adjusted for age, sex, racial identity, healthcare worker status, and partner's education

[a] Definition: Participants with at least one of smell/taste problems, shortness of breath or difficulty breathing, rash/blisters/welts, persistent cough, intermittent fever, muscle/chest pain, heart palpitations, mouth or tongue ulcers lasting ⩾4 weeks; participants reporting only psychiatric, cognitive, or neurological COVID-19-related symptoms lasting ⩾4 weeks were excluded (e.g., only fatigue, confusion/disorientation/'brain fog', memory issues, depression/anxiety/changes in mood, and/or headache).

[b] Multiple imputation was performed with fully conditional specification using 20 imputed datasets

[c] *P* trend analysis used indicator levels as a continuous variable.

© 2022 Wang S et al. *JAMA Psychiatry.*

eTable 8. Association of types of distress and risk of subsequent long COVID lasting more than 8 weeks among persons with a positive SARS-CoV-2 test during follow-up, April 2020 - November 2021, N=3,142

	Long COVID cases/positive SARS-CoV-2 cases (No. %)	Model adjusted for age, sex, racial identity, healthcare worker status, and partner's education
Probable depression (PHQ-2)		
No	520/1574, 33.0	1.0 [Reference]
Subclinical symptoms	506/1174, 43.1	**1.30 (1.15-1.47)**
Yes	190/387, 49.1	**1.48 (1.25-1.75)**
P trend[a]		**<.001**
Probable anxiety (GAD-2)		
No	373/1211, 30.8	1.0 [Reference]
Subclinical symptoms	535/1286, 41.6	**1.35 (1.18-1.55)**
Yes	311/642, 48.4	**1.58 (1.36-1.85)**
P trend[a]		**<.001**
Worry about COVID		
Not at all/not very worried	307/984, 31.2	1.0 [Reference]
Somewhat worried	673/1671, 40.3	**1.26 (1.10-1.45)**
Very worried	239/485, 49.3	**1.56 (1.32-1.85)**
P trend[a]		**<.001**
Perceived stress		
Q1, 0-2 points, (least)	150/506, 29.6	1.0 [Reference]
Q2, 3-4 points	139/425, 32.7	1.12 (0.89-1.42)
Q3, 5-6 points	116/309, 37.5	**1.32 (1.03-1.68)**
Q4, 7-14 points, (most)	164/346, 47.4	**1.72 (1.36-2.18)**
P trend[a]		**<.001**
Loneliness		
Hardly ever	144/457, 31.5	1.0 [Reference]
Less than some of the time	223/680, 32.8	1.04 (0.84-1.28)
Some of the time or often	201/443, 45.4	**1.46 (1.18-1.81)**
P trend[a]		**.003**

Note. PHQ-2 = 2-item Patient Health Questionnaire-2; GAD-2 = 2-item Generalized Anxiety Disorder. As perceived stress and loneliness were collected only in participants who were not active healthcare workers (N=1,628), risk ratios for number of types of distress were calculated only in these participants.
[a]*P* trend analysis used indicator levels as a continuous variable.

© 2022 Wang S et al. *JAMA Psychiatry.*
